# Intralimb coordination as a sensitive indicator of motor-control impairment after spinal cord injury

**DOI:** 10.3389/fnhum.2014.00148

**Published:** 2014-03-17

**Authors:** Lea Awai, Armin Curt

**Affiliations:** Spinal Cord Injury Center, Balgrist University Hospital, University of ZurichZurich, Switzerland

**Keywords:** spinal cord injury, human, gait, intralimb, coordination, categorization, cyclogram

## Abstract

**Background**: Recovery of walking function after neurotrauma, e.g., after spinal cord injury, is routinely captured using standardized walking outcome measures of time and distance. However, these measures do not provide information on possible underlying mechanisms of recovery, nor do they tell anything about the quality of gait. Subjects with an incomplete spinal cord injury are a very heterogeneous group of people with a wide range of functional impairments. A stratification of these subjects would allow increasing sensitivity for hypothesis testing and a more targeted treatment strategy.

**Methods:** The gait of incomplete spinal cord injured subjects was compared to healthy control subjects by analyzing kinematic data obtained by a 3-D motion capture system. Hip–knee angle-angle plots (cyclograms) informed on the qualitative aspect of gait and the intralimb coordination. Features of the cyclogram, e.g., shape of the cyclogram, cycle-to-cycle consistency and its modulation due to changes in walking speed were discerned and used to stratify spinal cord injured subjects.

**Results:** Spinal cord injured subjects were unable to modulate their cyclogram configuration when increasing speed from slow to preferred. Their gait quality remained clearly aberrant and showed even higher deviations from normal when walking at preferred speed. Qualitative categorization of spinal cord injured subjects based on their intralimb coordination was complemented by quantitative measures of cyclogram shape comparison.

**Discussion:** Spinal cord injured subjects showed distinct distortions of intralimb coordination as well as limited modulation to changes in walking speed. The specific changes of the cyclograms revealed complementary insight in the disturbance of lower-limb control in addition to measures of time and distance and may be a useful tool for patient categorization and stratification prior to clinical trial inclusion.

## INTRODUCTION

The most obvious impairment after spinal cord injury (SCI) is the complete or partial loss of lower-limb motor function, clinically assessed as decreased walking speed and alterations in time–distance measures (e.g., step length, step frequency, double-, and single-limb support phase; Krawetz and Nance, 1996; Pepin et al., 2003). These parameters are often used to monitor recovery and to capture locomotor capacity, but they lack the ability to unveil underlying neurological mechanisms (Dobkin et al., 2006; Tamburella et al., 2013). However, the quality of walking, especially the ability to coordinate lower-limb segments and joints, represents aspects of gait that complement the information about walking capacity and additionally provides insights into underlying mechanisms. A clinical inventory for gait quality assessment, relying on a subjective evaluation of SCI walking by a trained therapist has been developed (Field-Fote et al., 2001). Yet, it is not easy to quantify and scale gait quality and even compare it across different neuromotor disorders (Robinson and Smidt, 1981). The latter may be one of the reasons for the limited amount of published studies performing quantitative kinematic analysis of gait quality. Previous studies in healthy people have shown that, irrespective of walking speed, lower-limb segments are controlled interdependently as quantified by the co-variation of the elevation angles (Borghese et al., 1996; Grasso et al., 1998; Lacquaniti et al., 1999). This constraint in multi-segmental coordination suggests an underlying rule of motor control ensuring secure upright locomotion. The controlled behavior of multiple segments subserves the ultimate goal of controlling limb endpoints, which is believed to be controlled on different levels within very restricted boundaries. At the cost of aberrant muscle-activity pattern, the endpoint is controlled in a way that its trajectory shows very little variability in healthy individuals and gets closer to normal during training in iSCI subjects (Winter, 1992; Ivanenko et al., 2003). Compared to elevation angles, joint angles seem to be more variable and less reproducible (Borghese et al., 1996; Grasso et al., 1998). Lower-limb kinematics (i.e., hip-, knee-, and ankle-angle profiles) have been assessed in SCI (Abel et al., 2002; Gil-Agudo et al., 2011) and reveal aberrant and heterogeneous behaviors. But the simple description of joint angles during a gait cycle does not inform on intersegmental coordination. The intralimb coordination, measured as the simultaneous coordination of hip- and knee-angles, was found to be distorted in incomplete SCI (iSCI) and to be less amendable after locomotor training (Field-Fote and Tepavac, 2002; Nooijen et al., 2009). However, the degree of distortion has not been quantified and the measure of intralimb coordination has not been further analyzed. Also, the speed modulation of the intralimb coordination has not been considered, which might reveal information on underlying deficits. The aim of the present study was to characterize intralimb coordination of iSCI subjects qualitatively as well as quantitatively by means of hip–knee cyclograms. These measures may reveal different patterns and categories of walking impairment in iSCI subjects and may uncover mechanisms of lower-limb motor control. The latter findings may be applied to improve the stratification of patients for tailored (i.e., specific to the impairment) interventions to improve walking outcomes in acute as well as chronic iSCI.

## MATERIALS AND METHODS

### SUBJECTS

Incomplete SCI inclusion criteria: a diagnosed iSCI; age: 18 years or older; iSCI subjects need to be able to at least stand and walk without the assistance of another person (no manual leg movement by therapist). Exclusion criteria: Subjects suffering from neurological disorders other than SCI; gait impairments not caused by SCI. Healthy subjects with no neurological disorders or gait impairments served as the control group. The study was approved by the ethics committee of the Canton of Zurich, Switzerland. Participants gave their written informed consent. 19 iSCI subjects (4 female, 15 male; age: 50.0 ± 15.9 years; height: 172.6 ± 7.7 cm; weight: 75.8 ± 13.2 kg; Table 1) and 19 healthy control subjects (10 female, 9 male; age: 40.7 ± 13.7 years; height: 173.2 ± 9.3 cm; 68.9 ± 13.0 kg) were included in this study.

**Table 1:** Descriptive data of patients.

ID	Age (years)	Sex	Cause of SCI	Level of SCI	Disease progress	LEMS	Assistive device
01	73	Male	Traumatic	C5	Chronic	24.5	Wheeler
02	24	Male	Traumatic	C3	Chronic	24.5	–
08	60	Female	Spinal ischemia	T5	122 d	23.0	Wheeler
09	48	Female	Traumatic	T7	Chronic	15.0	One stick
10	39	Female	Spinal myelitis	C, T	46 d	19.0	Wheeler
12	65	Male	Hematoma	C6	Chronic	24.0	–
13	23	Male	Traumatic	C7	1 year	9.5	Wheeler
15	78	Male	Diverse	C3/4	50 d	22.5	Wheeler
16	60	Male	Spinal canalstenosis	T9/10	76 d	25.0	–
17	55	Male	Cervicalmyelopathy	C5	Chronic	19.5	–
18	63	Female	Epiduralabscess	T4	Chronic	23.0	Crutches
19	43	Male	Traumatic	C2	Chronic	24.0	Two sticks
20	61	Male	Disc prolapse	C2	Chronic	25.0	–
21	40	Male	Traumatic	C5	Chronic	22.5	–
22	64	Male	Spondylitis, abscess	T4	Chronic	23.5	–
23	32	Male	Traumatic	T11	75 d	8.0	Crutches
24	36	Male	Traumatic	L4/5	78 d	23.5	–
25	41	Male	Traumatic	C7	1 year	24.0	–
26	48	Female	Disc prolapse	T10	15 d	24.0	Two sticks

*LEMS = mean value of left and right lower extremity motor score (max = 25).*

### MATERIALS

Kinematic data was recorded using eight infrared cameras (T10, Vicon motion systems Ltd., Oxford, UK) at 200 Hz and two synchronized digital high-speed video cameras (pilot series, Basler AG, Ahrensburg, D) at 100 Hz. 16 reflective markers (16 mm diameter) were placed on bony landmarks according to the Vicon Plug-in Gait lower-body model. During treadmill (TM) walking pressure sensors underneath the TM belt recorded the force distribution of the footsoles at 120 Hz (Zebris FDM-T, zebris Medical GmbH, Isny im Allgäu, D). Kinematic data was recorded and post-processed using the Vicon Nexus Software (1.7.1). Trajectories were smoothed and gaps interpolated using Woltring’s cross-validatory quintic-spline routine with a mean squared error (MSE) of 10 mm^2^. Continuous data from ~20 consecutive gait cycles was cut into individual gait cycles and time-normalized using linear interpolation. Data from the pressure sensors underneath the TM belt was recorded by the same PC using the Zebris WinFDM-T software (02.01.01). Recordings were synchronized via a ±5 V trigger signal.

### PROTOCOL

Subjects walked barefooted both overground (OG) and on a TM, where walking speed could be controlled and varied. iSCI subjects were allowed to use assistive devices for OG walking or to hold handrails when walking on the TM. Subjects were first asked to walk OG to assess their preferred walking speed. On the TM, iSCI subjects were then asked to walk at a slow speed (0.5 km/h ≈ 0.14 m/s) and at preferred OG walking speed.

### OUTCOME VARIABLES

Approximately twenty gait cycles were analyzed per walking speed. The hip and knee angles were time-normalized to one gait cycle (500 samples) using linear interpolation. The intralimb coordination was illustrated by hip–knee cyclograms whose vertical and horizontal expansion corresponded to the maximal hip- and knee-range of motion [ROM (°)] during walking, respectively. The within-subject cycle-to-cycle consistency of these cyclograms was quantified using the angular component of coefficient of correspondence (ACC; Field-Fote and Tepavac, 2002). Inter-subject (within-group) variability of the cyclogram was assessed after translation of the centroids of cyclograms to the origin. The inter-subject variability was calculated as the cumulative ellipse area with half axes (a and b, see Eq. 1) corresponding to the between-subject standard deviation of hip- and knee-angles, respectively, for 20 equal bins of time-normalized cyclograms:

(1) $$Var_{n}=\overset{20}{\underset{i=1}{\sum }}\Pi *a_{n,i}b_{n,i}$$

Index *n* refers to subject number, *i* describe the bin number. The shape difference between two cyclograms was quantified as the square root of the sum of squared distances (SSD) after uniform scaling and translation of the cyclogram centroids to the origin:

(2) $$SSD_{j,k}=\sqrt{\underset{i}{\sum }{\left(\alpha_{j,i}‑\alpha_{k,i}\right)}^{2}+{\left(\alpha_{j,i}‑\alpha_{k,i}\right)}^{2}}$$

Equation 2 compares cyclogram *j* to cyclogram *k* where α and β correspond to the scaled and transformed hip- and knee-angles, respectively, at sample point *i*. Within-group SSD was calculated as the average SSD of every pairing of subjects within a group [mean of n^*^(n-1)/2 SSDs]. The between group SSD was calculated by comparing the mean cyclograms of two groups. Data analysis was performed using custom-written Matlab scripts (Matlab R2013a; The MathWorks Inc., Natick, MA, USA). A categorization based on the presence or absence of specific characteristics of the cyclogram shape was performed with the goal of stratifying patients into different groups of impairment. Within each group, iSCI subjects were additionally ranked according to the severity of alterations, resulting in a continuous ranking of iSCI subjects based on their cyclogram (lower ranking meaning worse cyclogram).

### STATISTICAL ANALYSIS

Normality of data distribution was tested using histograms and QQ plots. Non-normally distributed ACC values were compared using the non-parametric Kruskal–Wallis test and, if revealing significance, the following *post hoc* tests were applied: independent data was tested using a Mann–Whitney *U* test and a Wilcoxon signed rank test was employed for paired data samples. The significance level was corrected for using Bonferroni correction resulting in an α-value of 0.05/4 = 0.0125. In order to assess the relation between the qualitative cyclogram ranking and objective measures (i.e., walking speed, hip-, and knee-ROM) non-parametric Spearman correlation coefficient ρ (rho) was calculated. Matlab R2013a was used for all statistical analyses.

## RESULTS

Incomplete SCI subjects exhibited a mean preferred walking speed of 0.57 ± 0.32 m/s while control subjects preferably walked at 1.16 ± 0.15 m/s.

### PATIENT CATEGORIZATION

**FIGURE 1:**
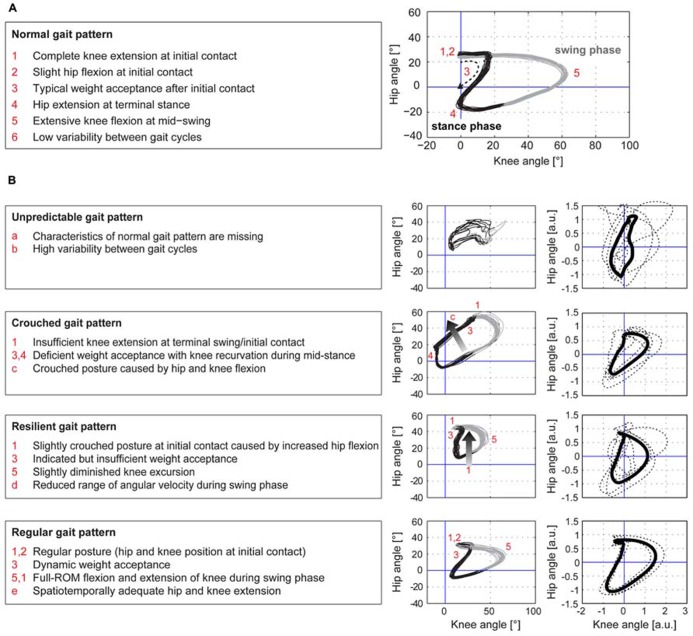
**Hip-knee cyclograms of healthy controls and iSCI subjects**. The hip–knee cyclogram of a healthy control subject with its typical characteristics is depicted in panel **(A)**. iSCI subjects were classified into four groups of impairment, group 1 (unpredictable gait pattern) being the most affected group while group 4 (regular gait pattern) showed normal cyclograms **(B)**. The criteria for each group are listed in the boxes on the left. The cyclograms in the left column show one representative example per group, the right column depicts the individual cyclograms of each iSCI subject within a group (dashed lines) and the group mean cyclogram (solid bold line) after translation of the cyclogram’s centroid to origin (zero) and uniform scaling.

**FIGURE 2:**
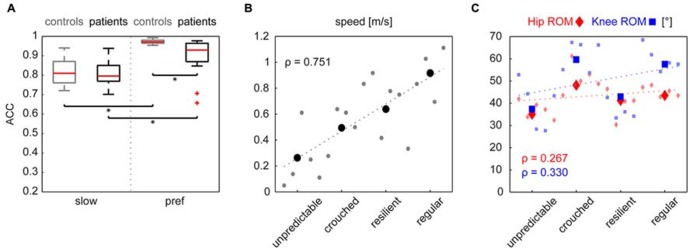
**(A)** The cycle-to-cycle shape consistency within a subject is quantified as the angular component of coefficient of correspondence (ACC). This value can attain values between 0 (no congruency between cycles) and 1 (complete shape congruency from cycle to cycle). Both groups increased their cyclogram consistency when changing from a slow to preferred walking speed. iSCI subjects only showed lower values at preferred walking speed. Statistical significance (*p* < 0.0125) is indicated by an asterisk. Panel **B** shows the correlation indicated by Spearman’s ρ of the cyclogram ranking to preferred OG walking speed and to hip- and knee-ROMs **(C)**. Little dots represent single values of individual subjects while the larger symbols represent the mean value per group.

The cyclograms of healthy control subjects at preferred speed exhibited typical characteristics that could be found in all subjects. These features are listed in the box on the left of **Figure 1A**. iSCI subjects showed one or more alterations of certain properties of the cyclogram and depending on the degree of deviation were classified into four groups of impairment (**Figure 1B**). The specific alterations of cyclogram characteristics are described in the boxes on the left. Accordingly, four iSCI subjects ended up in group 1 (unpredictable gait pattern), six iSCI subjects qualified for group 2 (crouched gait pattern), five iSCI subjects were classified into group 3 (resilient gait pattern), and finally four iSCI subjects exhibited a regular gait pattern (group 4). Correlation analysis between the cyclogram ranking and preferred OG speed, hip-ROM, and knee-ROM revealed a high correlation between preferred speed and cyclogram ranking (Spearman’s ρ = 0.751; **Figure 2B**) while both hip- and knee-ROM were relatively indifferent to the quality of the cyclogram (Spearman’s ρ = 0.267 and 0.330, respectively; **Figure 2C**).

### INTRALIMB COORDINATION

**FIGURE 3:**
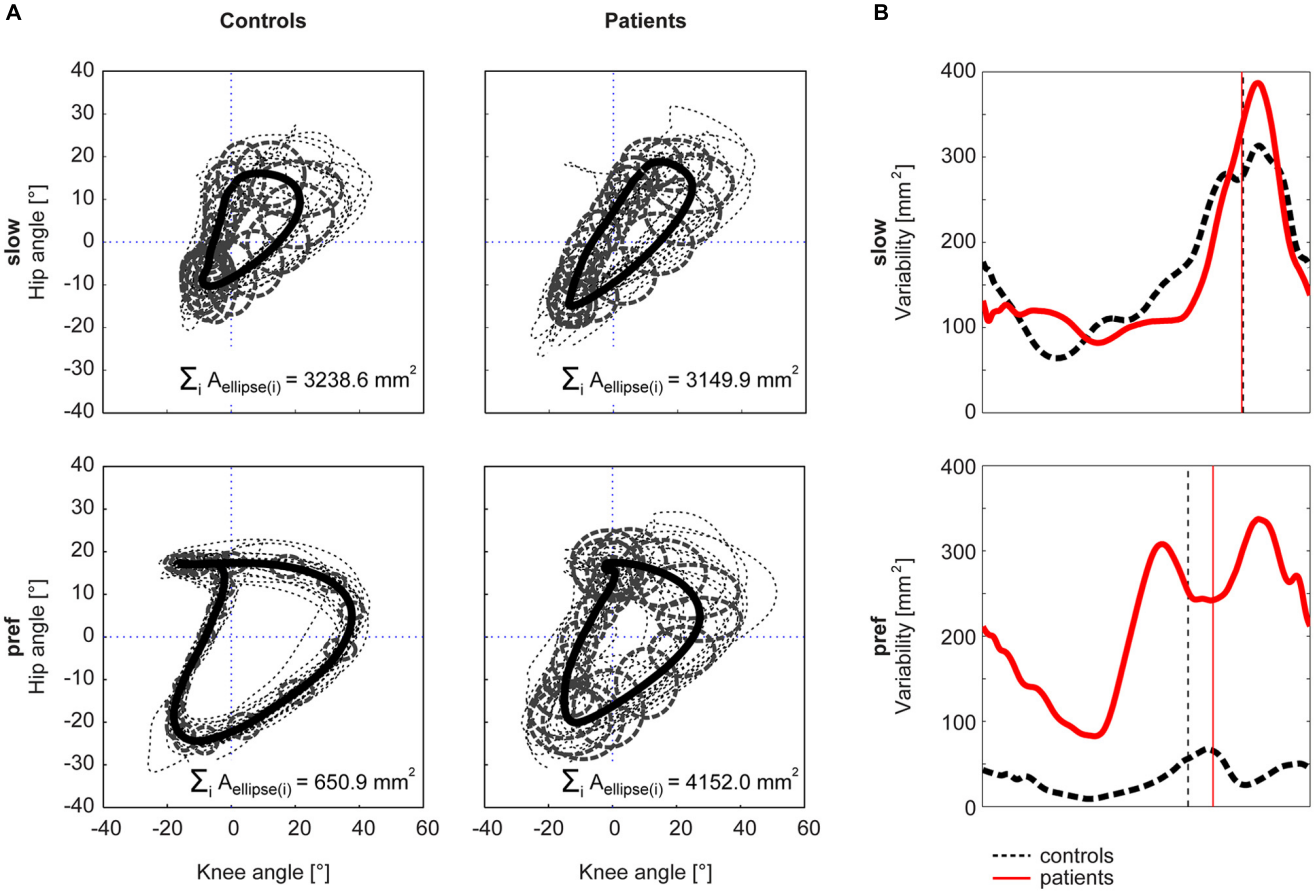
**Within-group cyclogram variability**. The variability of the cyclogram between subjects within a group was quantified by the cumulated elliptic area for 20 bins per gait cycle **(A)**. The half axes of the ellipse correspond to the between-subject standard deviation of the hip- and knee-angles, respectively. **(B)** shows the course of the variability at every time point of a gait cycle. The control group is represented by a black dashed line, iSCI group is depicted in red. The vertical lines mark the time point of toe-off.

**FIGURE 4:**
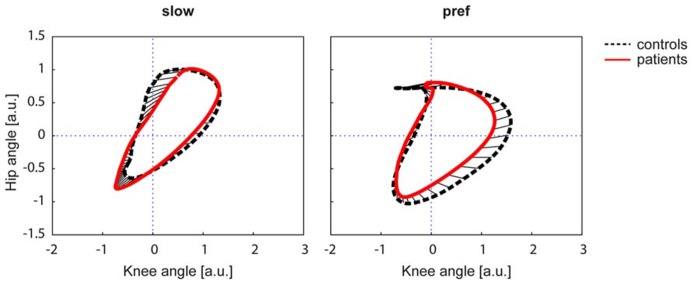
**The square root of the sum of squared distances (SSD) calculated the difference in shape of two figures**. The cyclograms are shown after translation of the centroid to origin (zero) and uniform scaling. The control group is represented by a black dashed line, iSCI group is depicted in red. The straight black lines indicate the deviation of the two figures. a.u. = arbitrary units.

The Kruskal–Wallis test revealed significant group differences in non-normally distributed ACC (χ^2^: 59.59, *p* < 0.001). At the slow walking speed (0.5 km/h) the cyclograms of healthy subjects were similarly distorted and irregular as iSCI subject’s cyclograms (**Figure 3A**). In line with this, the *post hoc* test revealed that the ACC did not differ between the two groups at the slow speed (Mann–Whitney *U* test: *p* = 0.840; **Figure 2A**). In contrast, control subjects normalized their cyclogram configuration to a very uniform shape between subjects when walking at preferred speed (**Figure 3A**). Likewise, the ACC was higher at preferred speed compared to the slow speed (Wilcoxon signed rank test: *p* < 0.001). iSCI subjects were also able to increase their ACC from slow to preferred speed (Wilcoxon signed rank test: *p* = 0.005), but their value was still significantly lower compared to healthy subjects (Mann-Whitney *U* test: *p* < 0.001). In contrast to control subjects, iSCI subject’s cyclograms did not converge toward normal and were still very heteromorphic. The within-group cyclogram variability during a gait cycle was similar in healthy control subjects and in iSCI subjects at the slow walking speed (**Figure 3B**). The cumulative variability for 20 equal time bins was 3238.6 mm^2^ in controls and 3149.9 mm^2^ among iSCI subjects. However, at preferred walking speed, control subjects showed a remarkably lower variability than iSCI subjects. The cumulative variability at preferred speed for 20 time bins among control subjects was 650.9 mm^2^ while it was even higher at preferred speed compared to the slow speed in iSCI subjects (4152.0 mm^2^). The SSD, representing the amount of shape difference after uniform scaling and translation (Table 2), was smaller within the healthy control group (SSD = 6.44) compared to iSCI subjects (SSD = 32.96) at preferred speed, but showed no difference at the slow speed (healthy: SSD = 14.00, iSCI: SSD = 11.74; **Figure 4**). The between-group shape difference was identical at the slow speed (SSD = 6.49) as at preferred speed (SSD = 6.46). Table 2 also shows the values of cyclogram shape comparison between each of the four groups of iSCI subjects compared to the control group. At preferred speed, the difference to normal shape according to the SSD coincided with the qualitative categorization of the cyclogram configuration.

**Table 2:** Cyclogram-shape differences.

Group comparison	SSD	
	Slow	Preferred
Within control group	14.00	6.44
Within patients	11.74	32.96
Between controls and patients	6.49	6.46
Between group 1 and controls	9.02	13.64
Between group 2 and controls	7.20	8.86
Between group 3 and controls	9.77	5.46
Between group 4 and controls	4.22	2.73

*SSD = square root of sum of squared distances. The within-group SSD was calculated by comparing the cyclograms of subjects each by each and then calculating the mean value. The between-groups SSD was calculated by comparing the mean cyclogram per group.
*

## DISCUSSION

The kinematic analysis of walking pattern in human iSCI allows for categorization of lower-limb control during walking while these qualitative readouts of gait performance are complementary to measures of walking distance and speed. While the latter measures are able to quantify the capacity of walking the qualitative scoring provides additional information regarding intralimb motor control. The consideration of these combined outcomes will be meaningful to better target treatment interventions and improve the stratification of most suitable patients for clinical trials.

### MODULATION OF INTRALIMB COORDINATION

Visual inspection and quantitative analysis of the cyclograms at a slow walking speed (i.e., 0.5 km/h) revealed that healthy control subjects exhibited similarly variable intralimb coordination as iSCI subjects. Speed increments in the two groups to preferred walking speed, however, induced distinct adaptations in cyclogram configuration. While effectively all control subjects gained a very regular and inter-subjectively uniform cyclogram at their preferred walking speed, iSCI subjects maintained considerable shape variability between subjects and their cyclograms remained different from the typical shape found in healthy subjects. The visually observed lack of cyclogram modulation in iSCI subjects was also made evident by the quantification of shape differences represented by the SSD. According to this value, the inter-subject shape difference within the iSCI group was bigger at preferred speed compared to the slow speed. The lack of shape assimilation is contrary to healthy subjects and might suggest that patient’s impairments become more pronounced when walking at preferred speed. In iSCI subjects with most severe shape deformations (group 1 with the highest cyclogram impairments and the lowest walking speed) the increase in speed even induced an increase in shape deformity. This increase in shape deformity did not occur randomly as the reproducibility of this aberrant shape (i.e., ACC) increased at preferred walking speed, indicating that iSCI subjects have learned and consolidated an aberrant walking pattern in a rather abnormal manner.

### NEURAL MECHANISMS

The lack of cyclogram modulation when increasing slow walking speed to preferred speed, at which locomotion is supposed to be more economic (Cotes and Meade, 1960) may be associated with a limited access to supraspinal control. We deliberately refer to the term “supraspinal”, including corticospinal as well as other descending motor tracts (e.g., rubrospinal or reticulospinal). Several aspects of locomotion, e.g., reciprocal activation of lower-limb muscles, appear to be primarily controlled at a spinal level and depend less on supraspinal efforts as revealed in preclinical studies in animals (Barbeau and Rossignol, 1987; Duysens and Van de Crommert, 1998). In humans, however, the correct spatiotemporal coordination of the lower-limb muscles and joints during locomotion (complex postural control and segmental interplay) seems to be more dependent on intact and sufficient supraspinal control (Kuhn, 1950). Interestingly, in the present study, an individual iSCI subject was not able to normalize his or her cyclogram when increasing the walking speed from slow to preferred speed despite increasing supraspinal neural drive (Bachmann et al., 2013). However, those iSCI subjects with a higher preferred OG walking speed, probably reflecting greater access to supraspinal speed-driving centers, showed a closer-to-normal cyclogram. The distinct input on walking control can also be found in Parkinson’s subjects, where the relation between step length and cadence with changes in speed is distorted [contrary to iSCI (Pepin et al., 2003)] while the hip–knee cyclogram retains a normal but small scaled shape (Morris et al., 1998, 2005). The distortion of cyclograms in iSCI subjects may not only originate from motor deficits, but may also be a consequence of impaired sensory feedback. Motor behavior is known to be affected in disorders associated with severe sensory impairment (Courtemanche et al., 1996; Manor and Li, 2009). Studies with deafferented subjects particularly revealed that the interjoint coordination was distorted while performing an upper-limb task (Sainburg et al., 1993). Even though skilled upper-limb movements depend more strongly on voluntary control compared to lower-limb movements (Nakajima et al., 2000; MacLellan et al., 2013), a deterioration of peripheral sensory feedback is sufficient to disrupt complex interjoint limb movements (Sainburg et al., 1993).

### IMPLICATIONS FOR TREATMENT AND CLINICAL TRIALS

The most widely used categorization scheme for SCI is the American Spinal Injury Association (ASIA) Impairment Scale (AIS). This scale is of rather low resolution and does not well distinguish patients upon walking abilities. In the present iSCI-subject sample the majority was classified as AIS D but ranged from AIS C/D to even AIS A (a subject with lesion level below T11). The categorization of iSCI subjects based on the cyclogram can complement the ASIA assessments and may serve several goals. From a therapist’s point of view treatment interventions aiming at improving locomotion may become better tailored to the patient’s walking impairment. Depending on the aim of training studies (e.g., improvement of quantitative measures and/or gait quality) different outcome measures should be chosen. Interventional studies may reveal that walking speed is improved while the gait quality remains impaired, or vice versa. Clinical trials investigating novel interventions (e.g., effect of drugs or cell transplantation) may have harmful or desired beneficial effects. By deploying the most sensitive assessment battery, differential effects may be discerned, and mechanisms of action may be recognized.

### LIMITATIONS

A sample size of 19 iSCI subjects is too low to be able to state whether the categorization based on the cyclogram quality is sufficient to stratify every iSCI subject. The categorization should be tested in a larger cohort, from which one could retrieve further quantitative measures that may serve as threshold values for future guidelines.

### CONCLUSION

In clinical routines walking ability is captured by walking speed and distance covered, but the quality of walking is frequently ignored or reported according to subjective rater evaluation. Yet, the present study shows that the intralimb coordination represented by the hip–knee cyclogram reveals reliable and sensitive information on walking capacity after iSCI and may be used to stratify patients prior to clinical trial inclusion. Further, the cyclogram with its quantifiable parameters may provide additional insights into gait control across different neuromotor disorders beyond those assessed by time-distance measures.

## Conflict of Interest Statement

The authors declare that the research was conducted in the absence of any commercial or financial relationships that could be construed as a potential conflict of interest.
